# The effects of low HER2 expression on survival in patients with metastatic breast cancer treated with CDK 4/6 inhibitors: a multicenter retrospective study

**DOI:** 10.1007/s10549-024-07291-0

**Published:** 2024-03-25

**Authors:** Murad Guliyev, Gülin Alkan Şen, İlkay Gültürk, Nargiz Majidova, Goncagül Akdağ, Ali Ahadzade, Hande Turna, Nebi Serkan Demirci

**Affiliations:** 1 Division of Medical Oncology, Department of Internal Medicine, Cerrahpaşa Faculty of Medicine, İstanbul University-Cerrahpaşa, Istanbul, Turkey; 2 Department of Medical Oncology, Bakirkoy Dr. Sadi Konuk Training and Research Hospital, Istanbul, Turkey; 3 Division of Medical Oncology, Department of Internal Medicine, Marmara University, Istanbul, Turkey; 4 Department of Medical Oncology, Kartal Dr. Lütf Kirdar City Hospital, Health Science University, Istanbul, Turkey; 5 Department of Internal Medicine, Cerrahpaşa Faculty of Medicine, İstanbul University-Cerrahpaşa, Istanbul, Turkey

**Keywords:** Breast cancer, Ribociclib, Palbociclib, HER2-low

## Abstract

**Purpose:**

Endocrine therapy (ET) in combination with CDK 4/6 inhibitors (CDK 4/6i) is the standard treatment modality for hormone receptor (HR)-positive and HER2-negative metastatic breast cancer (mBC). There is uncertainty about the prognostic and predictive value of HER2-low status and whether HER2-low BC is an individual biologic subtype. In this study, we aimed to investigate the prognostic effect of HER2 expression status on survival in mBC patients treated with first-line ET plus CDK 4/6i.

**Methods:**

This multicenter retrospective study included patients with HR + /HER2-negative mBC cancer who were treated with first-line CDK 4/6i in combination with ET from January 2016 to March 2023. Patients were divided into two groups (HER2-low and zero), and survival and safety analyses were performed.

**Results:**

A total of 201 patients were included in this study; of these, 73 (36.3%) had HER2-low disease and 128 (63.7%) had HER2-zero. There were 135 patients (67.2%) treated with ribociclib and 66 (32.8%) with palbociclib. Most of the patients (75.1%) received aromatase inhibitors as combination-endocrine therapy. Baseline characteristics were similar between the two groups. The median follow-up was 19.1 months (range: 2.5–78.4). The most common side effect was neutropenia (22.4%). The frequency of grade 3–4 toxicity was similar between the HER2-zero and low patients (32% vs 31.5%; *p* = 0.939). Visceral metastases were present in 44.8% of patients. Between the HER2-low and zero groups, median PFS (25.2 vs 22.6 months, *p* = 0.972) and OS (not reached vs 37.5 months, *p* = 0.707) showed no statistically significant differences.

**Conclusion:**

The prognostic value of HER2-low status remains controversial. Our study showed no significant effect of HER2 low expression on survival in patients receiving CDK 4/6i plus ET.

**Supplementary Information:**

The online version contains supplementary material available at 10.1007/s10549-024-07291-0.

## Introduction

According to the 2020 data from the global cancer database, female breast cancer (BC) surpassed lung cancer and was the most common type of cancer in the world [1]. BC is also one of the leading causes of death among women. Although mBC is still not a curable disease, new therapy agents have led to encouraging results in survival. BC is a very heterogeneous disease, consisting of different biological subtypes with different prognoses. Gene expression profiling studies have shown that breast cancer consists of six main intrinsic subgroups (luminal A, luminal B, basal-like, HER2-enriched, normal breast-like, and claudin-low) [2, 3]. The treatment decision in mBC is generally based on the hormone receptor (HR) and human epidermal growth factor receptor 2 (HER2) receptor results.

HER2 overexpression is observed in approximately 15–20% of all breast cancer patients and is known to be associated with a poor prognosis before the era of anti-HER2 therapy [4]. HER2 low expression is present in approximately 45–55% of HER2-negative mBC patients [5]. Patients with HER2 scores of + 1 and + 2 by immunohistochemistry (IHC) and without HER2 gene amplification by in situ hybridization (ISH) are defined as having HER2-low disease [6]. There is some evidence suggesting that patients with low HER2 expression should be evaluated as a separate subgroup of BC [7]. The different biological characteristics of HER2-low disease or its direct effects on prognosis are not fully known, and there is no consensus on this issue. Although positive results were not obtained with anti-HER2 treatments in this subgroup until recent years, the positive results of trastuzumab-deruxtecan, a new drug-antibody conjugate, on survival in HER2-low disease shown in the DESTINY 04 Breast study have led to increased interest in this subgroup [8].

According to the results of the PALOMA [9–11], MONARCH [12, 13], and MONALEESA [14–16] studies published in recent years, the use of cyclin-dependent kinase 4 and 6 inhibitors (CDK 4/6i) plus ET has become the first-line standard treatment in most patients with HR-positive, HER2-negative BC [17]. It is known that there is crosstalk between the HER2 and HR pathways, and HER2 expression can be modulated even in the absence of gene amplification [18, 19]. The presence of this bidirectional crosstalk between HR and HER2 may be one of the reasons for resistance to ET. In some studies conducted among BC patients receiving neoadjuvant chemotherapy, it was observed that pathological complete response rates were lower in the HER2-low group compared to HER2-zero [20]. Among patients with HR + /HER2-negative mBC, the effects of low HER2 expression on the efficacy of CDK 4/6i are not yet fully known, and there are limited and controversial data in the literature.

In our multicenter study, we aimed to investigate the effects of HER2 status on progression-free survival (PFS) and overall survival (OS) in HR + /HER2-negative mBC patients using CDK 4/6i as the first-line treatment.

## Materials and methods

### Patients

Patients who were diagnosed with mBC and treated with first-line CDK 4/6i in combination with ET combination therapy between January 2016 and March 2023 in four different oncology clinics were evaluated retrospectively. Baseline data were extracted from databases and medical records. Inclusion criteria were: 1) histopathologically confirmed BC; 2) radiologically proven metastatic disease; 3) HR + /HER2-negative disease proven by IHC staining and ISH; 4) ET plus CDK 4/6i used in the first line. Exclusion criteria were defined as: 1) Less than 3 cycles of CDK 4/6i treatment; 2) Patients who discontinued follow-up in our clinics; 3) Missing treatment response assessment.

HR and HER2 receptor evaluations were performed in each center's own pathology laboratory. In accordance with the American Society of Clinical Oncology/College of American Pathologists (ASCO/CAP) guidelines, patients with IHC + 1, + 2 and no gene amplification by ISH were considered to have HER2-low disease. ER or PR positivity was determined to be > 10% in IHC. Receptor results were based on biopsy results either from the primary tumor or metastatic site (whichever was available). Patients were divided into two groups: HER2-low and HER2-zero. If HER2-low expression was detected in one of the biopsy results obtained from the primary tumor or metastatic area, the patient was included in the HER2-low group. Age at diagnosis, menopausal status, detection of metastatic disease in denovo or recurrent disease, hormone levels, number of metastatic sites, presence of visceral or non-visceral metastasis, which ET they received with CDK 4/6i, grade ≥ 3 toxicities were evaluated. PFS and OS differences between the two groups, treatment-related toxicities and dose reduction rates were analyzed. Objective responses to treatment could not be evaluated because the study was multicentre and the evaluation could not be performed optimally according to Response Evaluation Criteria in Solid Tumors (RECIST) criteria.

This study was approved by the local ethics committee for clinical trials (date: July 12, 2023 and number: E-83045809–604.01.01–731418), and the need for informed consent was waived because of the retrospective nature of this study.

### Statistical analysis

The characteristics of the patients were compared with the Fisher or Chi-squared test for categorical data and a t-test for continuous data. OS was defined as the time from the initiation of CDK 4/6i until death from any cause. PFS was defined as the time from the initiation of CDK 4/6i to the date of radiological progression or death from any cause. No-event patients were censored at the end of the last follow-up. Survival curves were estimated by the Kaplan–Meier method and compared with the log-rank test. Cox regression was used to analyze the hazard ratios for PFS and OS. Statistical tests were two-sided, and a p-value less than 0.05 was considered statistically significant. The statistical analyses were conducted using SPSS version 26.

## Results

### Characteristics of patients

Our study included 201 patients. All patients were female. There were 128 (63.7%) patients in the HER2-zero group and 73 (36.3%) patients in the HER2-low group. The median follow-up time was 19.1 months (range: 2.5–78.4 months). The median age was 55 years (range: 26–82). The median age was similar between the two groups (54 vs 58 years, p = 0.702). The baseline characteristics of the patients are displayed in Table 1. Upon examination of histological subgroups, it was shown that the invasive ductal carcinoma subtype accounted for the majority, comprising 76.6% of cases. Approximately 2/3 of the patients had received ribociclib treatment. The numbers of patients treated with ribociclib or palbociclib were similar between the HER2-zero and low groups. All patients had received CDK 4/6i plus ET as a first-line treatment in the metastatic setting. Most patients (66.7%) were postmenopausal at the time of diagnosis. Although 120 patients (59.7%) had metastatic disease at the time of diagnosis, 81 patients (40.3%) developed metastatic disease at the time of recurrence, and the median time to reccurrence is 62.6 months (range: 12.6–278.7 months). There was no significant difference between the median ER (%) levels between the two groups. The majority of patients (75.1%) received an aromatase inhibitor (AI) treatment in combination with CDK 4/6i.

**Table 1:** Baseline characteristics of patients

Variable	Total	HER2-zero	HER2-low	*p* value
	(*n* = 201)	(*n* = 128)	(*n* = 73)	
Age (years), median (range)	55 (26–82)	54 (26–82)	58 (27–82)	0.702
Age (years), *n* (%)				
< 65	143 (71.1)	92 (71.9)	51 (69.9)	0.762
≥ 65	58 (28.9)	36 (28.1)	22 (30.1)	
Histology, *n* (%)				
IDC	154 (76.6)	97 (75.8)	57 (78.1)	0.212
ILC	20 (10)	16 (12.5)	4 (5.5)	
IDC+ILC	27 (13.4)	15 (11.7)	12 (16.4)	
CDK 4/6 inhibitors, *n* (%)				
Ribociclib	135 (67.2)	87 (68)	48 (65.8)	0.748
Palbociclib	66 (32.8)	41 (32)	25 (34.2)	
Menopausal status, *n* (%)				
Premenopausal	67 (33.3)	43 (33.6)	24 (32.9)	0.917
Postmenopausal	134 (66.7)	85 (66.4)	49 (67.1)	
Metastatic disease status, *n* (%)				
Denovo	120 (59.7)	79 (61.7)	41 (56.2)	0.44
Recurrence	81 (40.3)	49 (38.3)	32 (43.8)	
ER (%), median (range)	90 (15-100)	92.5 (20-100)	90 (15-100)	0.598
Metastatic site, *n* (%)				
≤ 2	120 (59.7)	73 (57)	47 (64.4)	0.307
> 2	81 (40.3)	55 (43)	26 (35.6)	
Type of metastasis, *n* (%)				
Non-visceral	111 (55.2)	69 (53.9)	42 (57.5)	0.619
Visceral	90 (44.8)	59 (46.1)	31 (42.5)	
Endocrine therapy, *n* (%)				
AI	151 (75.1)	100 (78.1)	51 (69.9)	0.193
Fulvestrant	50 (24.9)	28 (21.9)	22 (30.1)	
Grade 3,4 toxicity, *n* (%)	64 (31.8)	41 (32)	23 (31.5)	0.939
Toxicity-related dose reduction, *n* (%)	49 (24.3%)	34 (26.6)	15 (20.5)	0.34

*HER* human epidermal growth factor receptor, *IDC* invasive ductal carcinoma, *ILC* invasive lobular carcinoma, *CDK* cyclin dependent kinase, *ER* estrogen receptor, *AI* aromatase inhibitors

Metastases were present in three or more anatomical sites in 120 patients (59.7%) and in one or two sites in 81 patients (40.3%). A significant proportion of the patients (44.8%) had visceral organ metastases. Visceral and non-visceral organ involvement was similar between the two groups. A total of 13 patients had brain metastases, 8 of whom were in the HER2-zero group and 5 in the HER2-low group. In 73 patients (36.3%), the diagnosis was confirmed by biopsy from the metastatic site. The distributions of sites are summarized in Table 2.

**Table 2:** Anatomic location of metastasis

Location	Patients, *n* (%)			*p* value
	Total (*n* = 201)	HER2-zero (*n* = 128)	HER2-low (*n* = 73)	
Bone	143 (71.1)	90 (70.3)	53 (72.6)	0.73
Lymph nodes	54 (26.9)	29 (22.7)	25 (34.2)	0.075
Lung	54 (26.9)	36 (28.1)	18 (24.7)	0.594
Liver	39 (19.4)	25 (19.5)	14 (19.2)	0.951
Central nervous system	13 (6.5)	8 (6.3)	5 (6.8)	0.868
Adrenal gland	6 (2.9)	3 (2.3)	3 (4.1)	
Pleura	3 (1.5)	2 (1.6)	1 (1.4)	
Jejunum	2 (1)	2 (1.6)	0	
Peritoneum	1 (0.5)	1 (0.8)	0	
Colon	1 (0.5)	0	1 (1.4)	
Stomach	1 (0.5)	1 (0.8)	0	
Skin	1 (0.5)	0	1 (1.4)	

*HER* human epidermal growth factor receptor

The evaluation of adverse events during treatment revealed that 64 individuals (31.8%) developed grade 3 or higher toxicity. Neutropenia was the most common adverse event (22.4%). Thrombocytopenia developed in 3 patients, anemia in 5 patients, QT prolongation in 5 patients, diarrhea in 2 patients, hepatitis in 2 patients, and nephropathy in 2 patients. Grade 4 toxicity (hepatitis and neutropenia) developed in two patients. In 49 (24.3%) patients, a dose reduction was performed due to toxicity. The dose reduction rate was similar between the HER2-zero and low groups (26.6% vs 20.5%,* p* = 0.34). There was no significant difference in toxicities between the HER2-zero and low groups (32% vs 31.5%; *p* = 0.939). There were no treatment-related mortalities or unexpected adverse events.

### Survival analyses

**Fig. 1:**
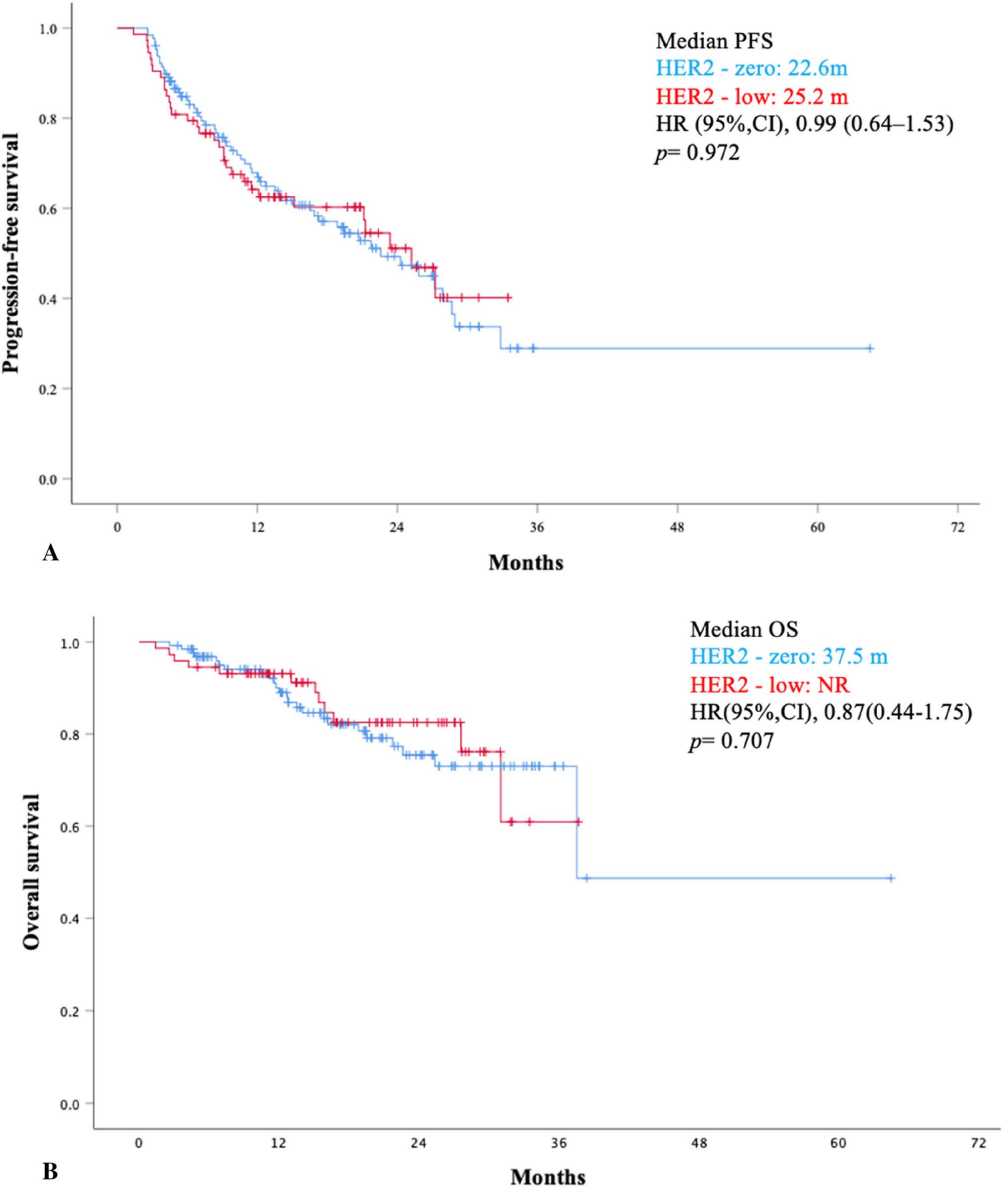
Progression-free survival (PFS) (**A**) and overall survival (OS) (**B**) in patients with HER2-low and HER2- zero

Patients with HER2-low disease had similar PFS when compared to patients with HER2-zero BC (median PFS: 25.2 vs 22.6 months; HR 0.99, 95% CI: 0.64–1.53, *p* = 0.972). The median OS in the HER2-low group was not reached (NR), but in the HER2-zero group it was seen to be 37.5 months. There was no statistically significant difference in median OS between the HER2-low and zero groups (HR 0.87, 95% CI: 0.44–1.75, *p* = 0.707). Survival analyses are shown in Fig. 1.

## Discussion

CDK 4/6i + ET combination therapy is the standard treatment regimen used in HR + /HER2-negative mBC, both in first-line and later-line settings. Low expression of HER2 is observed at a rate of ~ 60% among all BC patients [7]. In recent years, there has been data supporting the evaluation of HER2-low disease as a different entity [7, 20–22]. In a study by Schettini et al. evaluating 3689 patients, it was observed that low HER2 expression was more frequent in HR-positive disease than in triple-negative breast cancer (TNBC) (65.4% vs 36.5%, *p* < 0.001), and HER2-low patients were significantly associated with more nodal involvement and a larger primary tumor diameter compared to HER2-zero [5]. In another analysis of 523 patients analyzing genomic data, significant differences were observed between gene mutations seen in HER2-low and zero disease [23]. In this study, patients with low HER2 expression were more frequent in the HR-positive subgroup and had lower Ki67 expression levels than HER2-zero. In the same study, it was shown that PI3K-Akt signal pathway mutations were more frequent in the HER2-low group, and checkpoint factors, Fanconi anemia, p53 signaling, and cell cycle pathway mutations were more frequent in the HER2-zero group. After the use of antibody–drug conjugates such as trastuzumab deruxtecan in HER2-positive patients and the demonstration of its efficacy in HER2-low patients, interest in this subgroup has increased.

It has been observed that HER2-low disease is detected more frequently in HR-positive breast cancer patients than in the HR-negative group [20, 24]. Data have been published showing that low HER2 expression is associated with both lower pathological complete response rates (pCR) and worse survival in HR + BC patients receiving neoadjuvant chemotherapy. In a pooled analysis of four neoadjuvant chemotherapy trials involving a total of 2310 patients, it was observed that pCR rates were significantly lower in patients with HER2 low expression in the HR-positive subgroup compared to HER2-zero (17.5% vs 23.6%, *p* = 0.024), while there was no significant difference between the two groups in the HR-negative subgroup [20]. There are data supporting the existence of bidirectional crosstalk between HER2 and HR pathways as a potential mechanism of hormonal resistance and unfavorable outcomes on survival [18].

In our study, there were 36.3% of patients with low HER2 expression. Compared to previous studies [25–28], this rate was lower in our research. There could be several factors leading to this occurrence. One of them is the absence of a centralized pathology assessment. A recent study shows the lack of concordance between pathologists in distinguishing tumors with a HER2 score of 0 and those with a score of 1 + [29]. Another possible factor is that not all recurrent metastatic patients undergo a re-biopsy.

We investigated the effects of first-line ET in combination with CDK 4/6i treatment on OS and PFS in HER2-low and zero groups in patients with metastatic HR + /HER2-negative BC and we found no significant difference between the two groups in both OS and PFS. The literature has discrepancies in the findings of retrospective studies on this issue. Similar to the results in our study, Yildirim et al. conducted a multicenter retrospective analysis with 204 patients, and they found no significant impact of low HER2 expression on survival [30]. In another study conducted with a small group of patients, no significant difference was observed between objective response rates (ORR) in the HER2-low and zero patient groups [31].

Zattarin et al. [27] retrospectively evaluated 436 patients who received CDK 4/6i in the first line of the metastatic disease. In this study, it was observed that low HER2 expression was associated with poor OS and PFS, independent of other risk factors. In a retrospective analysis of 106 patients by Bao et al. [25], 77.3% of patients had HER2 low expression, and this subgroup had a shorter PFS (8.9 vs 18.8 months, *p* = 0.01) compared to HER2-zero patients. In this study, most patients (84.9%) were treated with palbociclib, and only half of the patients received first-line CDK 4/6 inhibitors.

It's not yet clear what the prognostic value of HER2 expression status is in HR + /HER2-negative mBC that is treated by CDK 4/6i plus ET. Studies on this subject in the literature are limited to retrospective analyses. Various factors might be responsible for the disparities shown in the findings of the studies. The assessment of HER2 status using different methodologies may be the most crucial among these factors. Another possible reason for the observed disparities among studies might originate from alterations to the patient group, including disparities in tumor-related parameters. A recent review evaluated nine retrospective analyses encompassing 2705 individuals [32]. The findings revealed a statistically significant increase in the risk of disease progression and death among patients with mBC who had low expression of HER2 and were treated with CDK 4/6 inhibitors.

Our study has several limitations that result from its retrospective design, which encompasses selection bias. Another limitation of our study is the short follow-up time for an optimal evaluation of survival outcomes*.* We consider the absence of centralization in the HER2 expression evaluation to constitute a significant limitation of our study that may have contributed to discrepancies in the results. According to the latest ASCO/CAP guideline, cases with tumor cell staining between 1 and 10% should be classified as ER-low positive [33]. However, in our country, insurance coverage for CDK 4/6 inhibitors is limited to patients with ER levels exceeding 10%. Therefore, patients classified as ER-low were not included in our study. Approximately 2 − 3% of breast cancers have been reported to be ER-low and there is limited data on the overall benefit of endocrine therapy in these patients [34]. Consequently, we suspect the exclusion of this patient subgroup from our study may have influenced our results.

In conclusion, our multicenter research suggests that low HER2 expression does not significantly affect survival outcomes in HR + mBC patients treated with first-line ET plus CDK4/6 inhibitors. Prospective analyses are needed to more accurately evaluate the effects of HER2 expression levels on treatment responses and survival in mBC patients treated with CDK 4/6 inhibitors.

### Supplementary Information

Below is the link to the electronic supplementary material.

Supplementary file1 (DOCX 176 KB)

## Data Availability

The datasets generated and/or analyzed during the current study are available from the corresponding author on reasonable request.
