# Supplementary material for: The effects of low HER2 expression on survival in patients with metastatic breast cancer treated with CDK 4/6 inhibitors: a multicenter retrospective study
Source: Breast Cancer Res Treat. 2024 Mar 25;205(3):633–40. doi: 10.1007/s10549-024-07291-0 (PMC11101584; doi:10.1007/s10549-024-07291-0)
Supplement: Supplementary file 1 — Supplementary file1 (DOCX 176 KB) [file 10549_2024_7291_MOESM1_ESM.docx]

**Supplemental Table 1. Baseline characteristics of patients**

| **Variable** | **Total**  (n=201) | **HER2-zero**  (n=128) | **HER2-low**  (n=73) | ***P* value** |
| --- | --- | --- | --- | --- |
| **Age (years), median (range)** | 55 (26-82) | 54 (26-82) | 58 (27-82) | 0.702 |
| **Age (years), n (%)**   - < 65 - ≥ 65 | 143 (71.1)  58 (28.9) | 92 (71.9)  36 (28.1) | 51 (69.9)  22 (30.1) | 0.762 |
| **Histology, n (%)**   - IDC - ILC - IDC+ILC | 154 (76.6)  20 (10)  27 (13.4) | 97 (75.8)  16 (12.5)  15 (11.7) | 57 (78.1)  4 (5.5)  12 (16.4) | 0.212 |
| **CDK 4/6 inhibitors, n (%)**   - Ribociclib - Palbociclib | 135 (67.2)  66 (32.8) | 87 (68)  41 (32) | 48 (65.8)  25 (34.2) | 0.748 |
| **Menopausal status, n (%)**   - Premenopausal - Postmenopausal | 67 (33.3)  134 (66.7) | 43 (33.6)  85 (66.4) | 24 (32.9)  49 (67.1) | 0.917 |
| **Metastatic disease status, n (%)**   - Denovo - Recurrence | 120 (59.7)  81 (40.3) | 79 (61.7)  49 (38.3) | 41 (56.2)  32 (43.8) | 0.44 |
| **ER (%), median (range)** | 90 (15-100) | 92.5 (20-100) | 90 (15-100) | 0.598 |
| **Metastatic site, n (%)**   - ≤ 2 - > 2 | 120 (59.7)  81 (40.3) | 73 (57)  55 (43) | 47 (64.4)  26 (35.6) | 0.307 |
| **Type of metastasis, n (%)**   - Non- visceral - Visceral | 111 (55.2)  90 (44.8) | 69 (53.9)  59 (46.1) | 42 (57.5)  31 (42.5) | 0.619 |
| **Endocrine therapy, n (%)**   - AI - Fulvestrant | 151 (75.1)  50 (24.9) | 100 (78.1)  28 (21.9) | 51 (69.9)  22 (30.1) | 0.193 |
| **Grade 3,4 toxicity, n (%)** | 64 (31.8) | 41 (32) | 23 (31.5) | 0.939 |
| **Toxicity-related dose reduction, n (%)** | 49 (24.3%) | 34 (26.6) | 15 (20.5) | 0.34 |

HER: human epidermal growth factor receptor; IDC: invasive ductal carcinoma; ILC: invasive lobular carcinoma; CDK: cyclin dependent kinase; ER: estrogen receptor; AI: aromatase inhibitors

**Supplemental Table 2.** Anatomic location of metastasis

| **Location** | **Patients, n (%)** | | | ***P* value** |
| --- | --- | --- | --- | --- |
|  | **Total** (n=201) | **HER2-zero** (n=128) | **HER2-low** (n=73) |  |
| Bone  Lymph nodes  Lung  Liver  Central nervous system  Adrenal gland  Pleura  Jejunum  Peritoneum  Colon  Stomach  Skin | 143 (71.1)  54 (26.9)  54 (26.9)  39 (19.4)  13 (6.5)  6 (2.9)  3 (1.5)  2 (1)  1 (0.5)  1 (0.5)  1 (0.5)  1 (0.5) | 90 (70.3)  29 (22.7)  36 (28.1)  25 (19.5)  8 (6.3)  3 (2.3)  2 (1.6)  2 (1.6)  1 (0.8)  0  1 (0.8)  0 | 53 (72.6)  25 (34.2)  18 (24.7)  14 (19.2)  5 (6.8)  3 (4.1)  1 (1.4)  0  0  1 (1.4)  0  1 (1.4) | 0.730  0.075  0.594  0.951  0.868 |

HER: human epidermal growth factor receptor

**A**

**
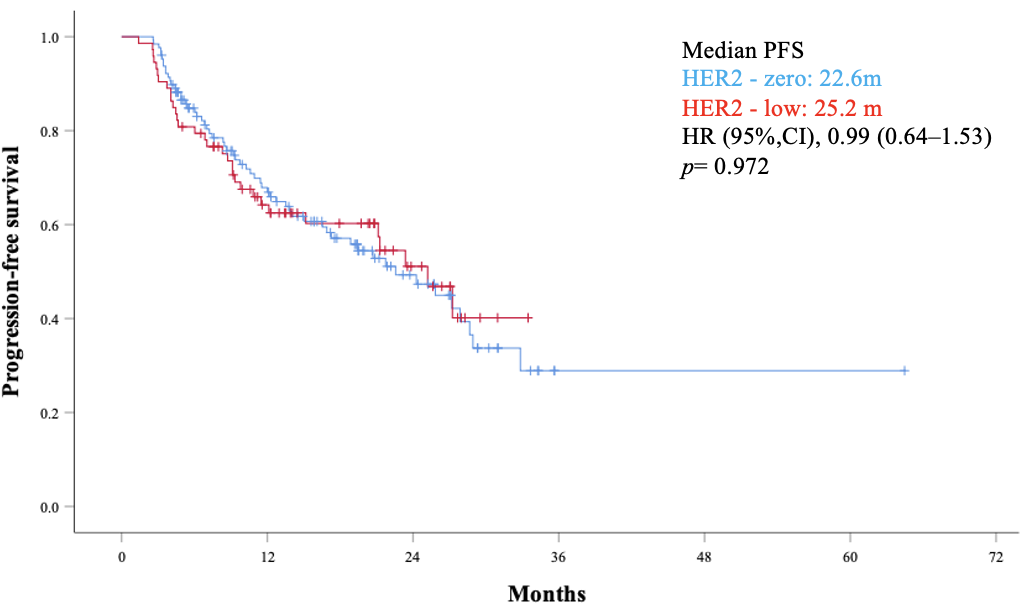
**

**B**

**
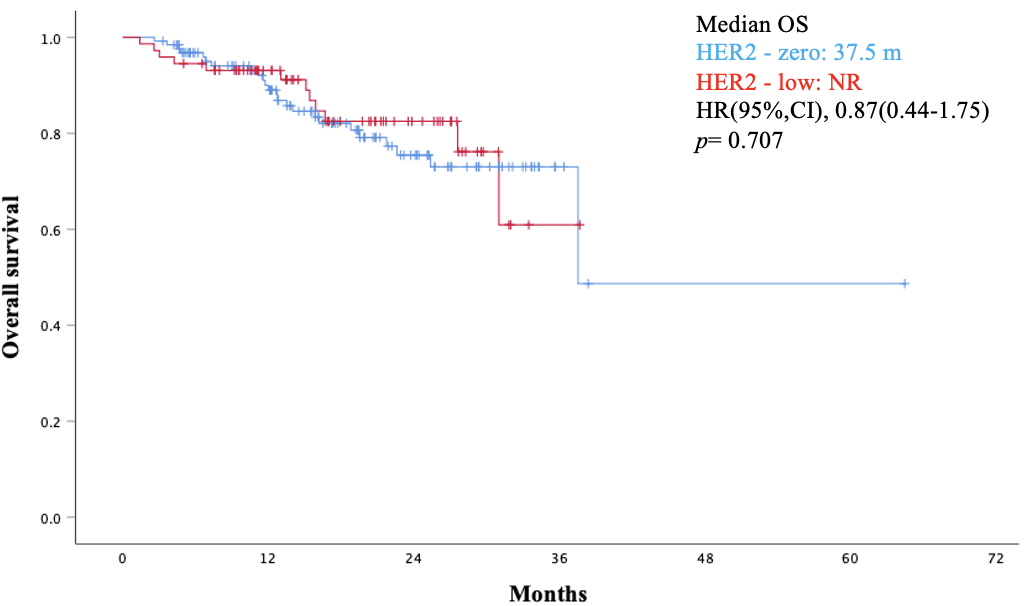
**

**Supplemental Figure 1.** Progression-free survival (PFS) (A) and overall survival (OS) (B) in patients with HER2-low and HER2-zero
